# Assessing the Impact of Frailty on Cognitive Function in Older Adults Receiving Home Care

**Published:** 2019-01-06

**Authors:** C Kleisiaris, EM Kaffatou, IV Papathanasiou, E Androulakis, S Panagiotakis, S Alvino, C Tziraki

**Affiliations:** 1 Nursing Department, Technological Educational Institute of Crete, Greece; 2 Nursing Department, Technological Educational Institute of Thessaly, Greece; 3 Faculty of Health and Welfare Professions, Technological Educational Institute of Athens, Greece; 4 Geriatric clinic, University General Hospital of Heraklion “PAGNI”, Greece; 5 Care Organization, SI4Life Liguria Region, Italy; 6 MELABEV-Community Club of Elders, and Hebrew University, Jerusalem Israel

**Keywords:** frailty, depression, cognitive decline, dementia, comorbidity, independence

## Abstract

It is commonly accepted that frailty and dementia-related cognitive decline are strongly associated. However, degree of this association is often debated, especially in homebound elders with disabilities. Therefore, this study aimed to investigate the association of frailty on cognitive function in older adults receiving homecare. A screening for frailty and cognitive function was conducted at 12 primary healthcare settings of the nationally funded program “Help at Home” in Heraklion Crete, Greece. Cognitive function and frailty were assessed using the *Montreal Cognitive Assessment* questionnaire and the *SHARE-f index*, respectively. *Barthel*-Activities of Daily Living and the *Charlson Comorbidity Index* were also used for the identification of disability and comorbidity, respectively. The mean age of the 192 participants (66% female) was 78.04 ± 8.01 years old. In depth-analysis using multiple linear regression, revealed that frailty was not significantly associated with cognitive decline (frail vs. non-frail (B′=−2.39, p=0.246) even after adjusting for depression and multi-comorbidity. Importantly, as protective factors for cognitive decline progression and thus dementia development, was scientifically correlated with annual individual income >4500 (B′=2.31, p=0.005) -poverty threshold-compared to those with <4500 and, higher education level as compared to Uneducated (B′=2.94, p=0.019). However, depression was associated with cognitive decline regardless of socioeconomic variables. In conclusion, our results suggest that health professionals caring for frail people with cognitive impairment, must focus on early recognition and management of depression.

## INTRODUCTION

Cognitive function refers to those mental processes that are crucial for the conduct of the intellectual and functional activities of daily living, leading to “neurocognitive disorders” including dementia and Alzheimer Disease [1]. Cognitive decline, including dementia, is strongly associated with co-morbidities in older adults such as systemic inflammation [2], diabetes mellitus type-2 [3]. coronary artery disease, arthritis and stroke. However, a large proportion of the variation of cognitive decline in late life is not due to common neurodegenerative pathologies [4], but rather psychosocial variables including low education, loneliness, gender, single marital status and frailty [5].

The prevalence of frailty in 65 years and older Greek community-dwelling population was found 14.7% and 44.9% at risk of frailty or pre-frail in accordance with the recent SHARE study criteria [6]. Frail older people present a faster and more-severe decline in cognitive function compared to pre-frail people [7]. Moreover, older adults have a higher change of developing Alzheimer Disease (AD) were previously observed [8 – 9]. In Greece, only a few studies have investigated the risk factors related to frailty, and no study was performed identifying the impact of frailty and its association with cognitive function among homebound elderly population.

In the present study, we aimed to examine the association of frailty on cognitive function, as well as other sociodemographic variables previously reported as risk factors in older adults aged 65+ receiving homecare.

## METHODOLOGY

### Study design

This cross-sectional study was conducted in 12 home care settings in a prefecture region of Heraklion, Crete, Greece, during a 6-month period (May to October 2017). Home care settings provide (free of charge) social, nursing, and medical care to their registered members. These members are mainly elderly people over 65 years old, with a disadvantaged social status and/or a poor family support. The registration criteria for the beneficiary elders are mainly “socioeconomic”, giving priority to people with disabilities who are not fully independent; need special care; are lonely or abandoned by family; and have insufficient financial resources. Following an invitation by the local municipality authorities, this study recruited these elderly members to participate in a door-to-door screening frailty and dementia program. A total of 744 elderly who are served by “Home Care” system in reference region of Heraklion were approached and 192 of them (response rate 38.8%) completed the study questionnaires and subsequently underwent frailty and dementia measurement after their informed consent.

### Study analysis

Frailty syndrome was assessed using the *SHARE-Frailty Instrument* that has been recently applied in 12 European countries [10]. SHARE-Fi was created via estimation of a discrete factor model based on five adapted phenotypic frailty items (i.e. grip strength and four self-reported items: fatigue, loss of appetite and/or eating less than usual, difficulties climbing stairs and/or walking 100 meters, and low level of physical activity). Practically, data was entered-into the web-based calculator and latent classes (non-frail, pre-frail and frail) was obtained for each sex. Specifically, the tool provides a continuous frailty score and enables classification into phenotypic frailty categories: non-frail, pre-frail and frail.

Cognitive decline was assessed using the *Montreal Cognitive Assessment scale (MoCA)* which was recently validated (sensitivity 0.82 and specificity 0.90) in a sample of 174 Greek patients with diagnosed parkinsonian dementia [11]. The final score is ranging 0–30, and value <26 is indicative of cognitive decline related to dementia development.

The independence in activities of daily living of an elderly population was assessed using the Greek version of the *Barthel-Activities of the Daily Living scale*. It is measures functional independence in the domains of personal care and mobility. Specifically, it measures what patient “does”, and not what patient “could do” [12]. The overall score ranges from 0–20, with lower scores indicating increased disability. In the present study, individuals that self-rated 0–10 were defined as “disabled” [13].

The Greek version of the *Geriatric Depression Scale (GDS)* was used to evaluate depression [14]. The short form of GDS is a standardized scale and consists of 15 closed-ended queries. A total score ranges 0–15. Higher scores indicate more depressive symptoms are present. In this study, we explored the effect of severe depression as self-rated with score 11 or more.

The *Charlson Comorbidity Index (CCI)* was used to assess the confounding effect of comorbidity risk on cognitive function. The CCI examines 15 pathological conditions (items) such as diabetes, hemiplegia, myocardial infarction, etc. [15]. Each of the items in the CCI is awarded several points, which are summed according to the answers about the patient. The index score is further calculated through the algorithm method. In the present study, we considered, based on the Charlson score, the following (ordinal) categories: 0–1 in the absence of comorbidity, 2–4 mild to moderate comorbidity, and ≥5 severe comorbidity following the suggestions of Huang and his colleagues [16].

*Homebound Status* of elders was also assessed for possible confounding effects. Homebound status refers to ability of person to leave or leaving the home due to its illnesses. In this study, homebound was considered to be a person that “left” the home at least once a week in the last month, “semi-homebound” about 2 times a week but with help and “non-homebound” about 2 times a week but without help [17].

An annual individual income (<4,500 euros) for the economic year of 2016 was considered as the “poverty threshold” of the participants according to the official socioeconomic data of the Hellenic Statistical Authority [18]. Demographic characteristics such as age, education, marital status, were also collected.

### Ethical considerations

The study was conducted as part of a commitment “*The role of homecare in the prevention of cognitive decline and frailty”* [19] as regards A3 Action Group “Lifespan Health Promotion & Prevention of Age Related Frailty and Disease”. Therefore, our commitment meets the written ethical approval by the leading organization - Nursing Department of Technological Educational Institute of Crete, Greece (Pr No 364, 16^th^ March 2017). All participants gave informed consent, after a detailed briefing by the researchers regarding the purpose and procedures of study, while underlining that their participation was voluntary.

### Statistical analysis

Categorical variables are summarized as frequencies and percentages (n, %), while continuous variables are presented as means and standard deviations. Shapiro-Wilk’s test, along with the visual overview of the corresponding histograms, normal Q-Q plots and box-plots where used in order to assess normality of continuous variables. Baseline differences between the examined groups were assessed with chi-square test, t-test or Mann-Whitney test, along with Monte Carlo simulations (10000 samples) when appropriate. Also, in order to investigate the impact of frailty on cognitive function crude linear regression models were performed, adjusting for potential confounding effects (age, gender, education, depression and comorbidity. Data analysis were presented using adjusted B confidence (β′), corresponding 95% confidence intervals (CI). A p-value<0.05 was considered statistically significant. Data were analyzed using the SPSS.

## RESULTS

### Demographic data

In table 1 shows the sociodemographic characteristics of the participants. The mean age was 78.04±8.01and 80% had no formal education. Diabetes melitus (32.6%) and Peripheral Vascular Disease (26.2%) were the most frequent chronic diseases according to CCI.

### Frequency of the main disorders

The prevalence of the cognitive decline (93.7%), frailty (45.9%), severe depression (14.7%), severe comorbidity (67.8%), disability (7.7%) and, homebound elders (25.1%) are presented in Table 2. Also, severe depression was significantly more frequently in frail elders in comparison to pre-frail and non-frail (19%, 13.2% and 0%) respectively – (data are not presented in table).

### Factors affecting cognitive function

In table 3, we present the association of frailty on cognitive decline as an independent variable including other risk factors focusing only on 179 patients with MoCA<26. Specifically, frail elders are expected to present a decreased cognitive function (−5.23 grades in MoCA, p=0.018) and thus cognitive decline compared to non-frail elders, suggesting that frailty and cognitive decline are significantly associated. Similarly, patients with severe depression are expected to present cognitive decline (−3.47, p=0.005) compared to patients with normal depression, whereas patients with severe comorbidity (CCI≥5) and male gender are not associated with significant cognitive decline (−1.12, p=0.233 and −1.39, p=0.132), respectively. On the other hand, it is observed that elders with annual personal income (>4.500 euros) and those with a greater educational level are significantly associated with better cognitive function (2.08, p=0.017 and 3.87, p=0.004) compared to those with lower annual income (<4.500) and Uneducated elders, respectively, suggesting that “to be rich” and “to be educated” are protective factors for cognitive decline or delay of dementia progression.

### The impact of frailty on cognitive function

In the table 4, we present the results of multiple linear regression (1^st^ model) adjusted for all independent variables exploring possible confounding effects. Importantly, in this model frailty and cognitive decline are not associated even though cognitive decline is observed (−5.23 grades in MoCA, p=0.367), suggesting that other risk factors may act as confounders in this association. Thus, we applied depth-analysis in order to control possible interactions in cognitive function. The results of this analysis are presented in Table 5. More specific, in both linear regression models (2^nd^ & 3^rd^) it is observed that frailty is still affecting cognitive function but only 2 grades in MoCA scale (−2.39, p=0.246 even after adjusting for depression and comorbidity. It is also observed that cognitive function is negatively affected by depression (−2.61, p=0.031) and age (−0.19, p=0.001), suggesting that cognitive function is decreased (−0.20 grade in MoCA per year). Also, a greater annual individual income (>4.500 euros) and higher educational level e (Bachelor/MSc/PhD) were associated with significantly higher cognitive function (2.30, p=0.005 and 2.94, p=0.004) respectively, suggesting that both higher individual income and higher educational level are protective factors for cognitive decline or delay the onset of dementia.

## DISCUSSION

To our knowledge, this the first study in Greece exploring the impact of frailty on cognitive function in elderly population receiving homecare. In this study, we present the preliminary results of our commitment to A3 Action Group of the European Innovation Partnership on Active and Healthy Ageing exploring the impact of frailty on cognitive decline related to dementia in older people focusing mainly on interactions could affect cognitive function. The strength of the present study is its homecare approach to the prevention of cognitive decline and frailty will help us to develop theory-driven and effective prevention strategies that are person-centered and accessible.

The main finding of the present study suggests that frailty in elders us associated with a higher probability of decreased cognitive function when compared to non-frail elders. However, there are some important cofounding variables. modifying this association. Particularly, when we adjusted for severe depression and comorbidity, the frailty-cognitive decline association was no longer significant, suggesting that not only frailty play a key role in the development of dementia, but also the presence of severe depression and cognitive decline due to aging alone (Table 5). We postulate therefore observed that cognitive decline/frailty association observed in our study, Is similar to the recently published in Chinese older adults, reporting that there is an independent relation between frailty and cognitive decline [20]. Another possible explanation could be that frailty and dementia share psychobiological pathways for example, oxidative stress and protein misfolding and systems mechanisms as well as impaired repair (failures in chaperone proteins, autophagy) give rise to deficits at this level [21]. Moreover, finding from a cross-sectional study in Portugal [22] have shown that the contribution of comorbidity affects both clinical syndromes and possibly weakness and fatigue adults who also have higher prevalence of depressive symptoms [23]. Our main finding is also supported by the findings of recent survey in Chinese elderly, showing that the institutionalized elderly patients with depressive symptoms are at higher risk for the occurrence of the frailty syndrome [24]. In our study, 19% of frail people suffered from severe depression, while and no depression (0%) was seen in the elderly detected as non-frail.

As expected from previous studies, physiological againing alone is indepentlay associated ognitive decline [25]. In this study, there was significant reduction of cognitive function (0.20 grades per year). The current hypothesis, is that a systemic inflammation during the ageing process may play an important role in dementia progression [2]. It is importnt to emphasize that a large proportion of the variation of cognitive decline in late life is not due to common neurodegenerative pathologies [4 – 5], but rather due to several modified risk factors such as heavy smoking, unhealthy diet, and poorer glycaemic control during the life-course. These modibiable festyle factors are linked to an accelerated late-life cognitive decline [26 – 3].

We also found that higher educational level and annual individual income (>4.500 euros) appear to act as protective factors in cognitive decline and possibly also slow the progression of dementia. Indeed, several studies have reported that “socioeconomic disadvantage” is a risk factor for dementia as associated with poorer cognitive performance but not faster cognitive decline [27 – 28].

### Future implications

In Greece, both patients with dementia and frailty remain mostly into their homes and thus their care is mainly provided by their family members (family caregivers) and less by professionals (t trained) caregivers. On the other hand, there are no standards in the provisions of home-care as well as homecare is largely provided by the local Municipality Authorities (open care community centers for older people [KAPIs] and the “help at home” programme), taking into consideration that most of the health professionals in these sectors are not trained in assessement/evaluation of the early ddiagnosis of frailty and dementia symptoms. Therfore, the opportunity for early detection and management of frailty and cognitive decline are lost. It is obvious therefore that there is a growing awareness for innovation of the Primary Healthcare System in Greece particularly on education and training of health professionals mainly community nurses in professional skills and competencies related to the prevention of cognitive decline and frailty adapting person/home centered care [29]. In this context, it has been strongly recommended that interventions that instruct and inform frail older people in how and why to change their behavior, or support physical environment modifications, appeared to show promise for improving physical and cognitive function [30].

Despite the useful findings that would help translate research into practice, our results have certain limitations. The most important limitation is that the results are self-reports of the elderly and it is therefore obvious that our results may deviate from the actual diagnosissupported by trained health professionals.. In addition to the constraints, some research difficulties, such as the low response rate (38.8%), since a large part of the elderly either denied to participat or reluctance to participate in the survey or did not meet the criteria for participating, or they did not have the patience to complete the many scales that are particularly demanding in our screening program. However, this is usual bias in homecare original studies, and therefore prevalence rates (%) were used only as variables in the statistical analysis.

## CONCLUSION

Our data analysis suggests that health professionals caring older for adults in “home care” setting should be trained for early diagnosis, assessment, evaluation of elders at risk for frail and cognitive impairment. Since depression and life styles are also important co-variants that are modifiable, they should be part of the early diagnosis and screening of home bound elders. Thus, health personnel (geriatricians & nurses) working with elders at home should be trained in early recognition of these variants. It is crucial therefore to enhance the training and capacities of r community nurses to include early diagnosis management of depression, cognitive decline and frailty

## 











## Figures and Tables

**Table 1:** Demographic data of the participants (n=192)

	Mean ± SD, Median (IQR)	
**Age (years)**	78.04±8.01, 78.00 (12.00)	
	**ν**	**%**
**Gender**		
Men	65	34.0
Women	126	66.0
**Annual individual Income**		
<4500	96	50.3
>4500	95	49.7
**Educational level**		
Uneducated	154	80.6
Highschool	21	11.0
Bachelor	15	7.9
MSc/PhD	1	0.5
**Marital status**		
Unmarried	14	7.3
Married	56	29.3
Divorced/Widowed	121	63.4
**Most frequent comorbidities (CCI)** a		
Diabetes Mellitus (Type I or II)	62	32.6
Peripheral Vascular Disease	50	26.2
Connective Web Disease	47	24.6
Congestive Heart Failure	41	20.5

Abbreviation:

a according to Charlson Comorbidity Index

**Table 2:** Frequency of the disorders to be investigated (n=192)

	ν = 191	%
**Cognitive Function** a		
MoCA <26	179	93.7
MoCA ≥26	12	6.3
**Frailty**		
Frail	84	45.9
Pre-frail	91	49.7
Non-frail	8	4.4
**Depressiom**		
Severe (GDS 11+)	28	14.7
Mild (GDS 6–10)	82	42.9
Normal (GDS 0–5)	81	42.4
**Comorbidity** b		
Severe (CCI≥5)	124	67.8
Mild (CCI 2–4)	59	32.2
Normal (CCI 0–1)	0	0.0
**Independence** c		
Depentent (Barthel≤10)	14	7.7
Semi-dependent (Barthel11–14)	22	12.0
Independent (Barthel +15)	147	80.3
**Homebound status** d		
Homebound	48	25.1
Semi-homebound	29	15.2
Non-homebound	114	59.7

**Notes:**

a **MoCA<26** indicates cognitive decline;

b **Comorbidity** refers to the mean values of the CCI index and not to the actual number of illnesses;

c (**Barthel≤10** indicates disability or “disabled” patients);

d **Homebound status** refers to the ability of a person to leave or leaving the home during the last month due to its illnesses.

**Table 3:** Investigation of the impact of frailty and other independent variables on cognitive function (MoCA) [(n = 179]

Independent variables	B′ (s.e) a	95% CI b	T	p-value
**Frailty**				
Frail vs non frail	−5.23 (2.19)	(−9.57, −0.895)	−2.38	0.018
Pre-frail vs non-frail	−3.04 (2.19)	(−7.36, 1.29)	−1.38	0.168
**Depression** (GDS)				
Severe vs normal	−3.47 (1.23)	(−5.89, −1.04)	−2.81	0.005
Mild vs normal	−0.83 (0.89)	(−2.60, 0.95)	−0.92	0.359
**Comorbidity**				
Severe (CCI≥5) vs mild	−1.12 (0.94)	(−2.97, 0.73)	−1.19	0.233
**Independence** (Barthel)				
Dependent vs independent	−3.37 (1.58)	(−6.50, −0.24)	−2.13	0.035
Semi-dependent vs independent	−1.52 (1.35)	(−4.19, 1.15)	−1.12	0.262
**Homebound status**				
Homebound vs non-homebound	−3.36 (0.95)	(−5.25, −1.47)	−3.51	0.001
Semi-homebound vs non-homebound	−1.41 (1.16)	(−3.69, 0.880)	−1.21	0.226
**Cardiovascular diseases (CVDs)**				
Yes vs No	−1.75 (0.84)	(−3.40, −0.10)	−2.09	0.038
**Age (years)** ^C^				
	−0.28 (0.05)	(−0.38, −0.18)	−5.47	<0.001
**Gender**				
Male vs female	−1.39 (0.92)	(−3.21, 0.42)	−1.51	0.132
**Annual individual income**				
>4500 vs <4500	2.08 (0.86)	(0.37, 3.78)	2.41	0.017
**Educational level**				
High school vs Uneducated	3.87 (1.34)	(1.23, 6.52)	2.89	0.004
Bachelor/MSc/PhD vs Uneducated	5.85 (1.51)	(2.86, 8.84)	3.86	<0.001

**Abbreviations:** β′ confidence

a (**s.e**): standard error;

b **CI**: Confidence Intervals;

c **Age**: increase or decrease grades of MoCA per year;

**Note**: MoCA is controlled as deepened variable in this linear model meaning.

**Example**: In the relation *“Frail vs. non-frail”* it is expected reduction of MoCA score (−5.23 grades), this means that as lower scores as greater cognitive function.

**Table 4:** In-depth analysis for potential confounding effects on association between frailty and cognitive function (n = 179)

Independent variables	Linear regression model				1^st^ model			
	B (s.e)	95% CI	t	p-value	B (s.e)	95% CI	t	p-value
**Frailty**								
Frail vs non frail	−5.23 (2.19)	(−9.57, −0.895)	−2.38	0.018	−1.89 (2.09)	(−6.03, 2.24)	−0.90	0.367
Pre frail vs non frail	−3.04 (2.19)	(−7.36, 1.29)	−1.38	0.168	−1.24 (2.03)	(−5.26, 2.77)	−0.61	0.542
**Depressiom** (GDS)								
Severe vs normal	−3.47 (1.23)	(−5.89, −1.04)	−2.81	0.005	−2.06 (1.27)	(−4.58, 0.45)	−1.62	0.107
								
Mild vs normal	−0.83 (0.89)	(−2.60, 0.95)	−0.92	0.359	−0.94 (0.91)	(−2.74, 0.85)	−1.04	0.299
**Independence** (Barthel)								
Dependent vs independent	−3.37 (1.58)	(−6.50, −0.24)	−2.13	0.035	−1.37 (1.53)	(−4.41, 1.66)	−0.89	0.374
								
Semi-dependent vs independent	−1.52 (1.35)	(−4.19, 1.15)	−1.12	0.262	0.46 (1.29)	(−2.10, 3.03)	0.35	0.721
**Homebound status**								
Home-bound vs non-homebound	−3.36 (0.95)	(−5.25, −1.47)	−3.51	0.001	−0.49 (1.13)	(−2.73, 1.74)	−0.44	0.662
								
Semi-homebound vs non-homebound	−1.41 (1.16)	(−3.69, 0.880)	−1.21	0.226	0.21 (1.21)	(−2.18, 2.61)	0.17	0.860
**Cardiovascular diseases**								
Yes vs No	−1.75 (0.84)	(−3.40, −0.10)	−2.09	0.038	−1.23 (0.81)	(−2.87, 0.32)	−1.58	0.115
**Age (Years)**								
	−0.28 (0.05)	(−0.38, −0.18)	−5.47	<0.001	−0.19 (0.05)	(−0.29, −0.09)	−3.69	<0.001
**Income**								
>4500 vs <4500	2.08 (0.86)	(0.37, 3.78)	2.41	0.017	2.26 (0.78)	(0.71, 3.81)	2.87	0.005
**Education**								
Highschool vs Uneducated	3.87 (1.34)	(1.23, 6.52)	2.89	0.004	3.19 (1.26)	(0.70, 5.68)	2.53	0.012
Bachelor/MSc/PhD vs Uneducated	5.85 (1.51)	(2.86, 8.84)	3.86	<0.001	4.19 (1.44)	(1.35, 7.03)	2.91	0.004

**Example**: In the relation *“Frail vs. non-frail”* it is expected reduction of MoCA score (−1.89 grades), this also means that as lower scores as greater cognitive function

**Table 5:** Adjusted analysis for factors affecting cognitive function (n = 179).

Independent variables	2^nd^ model a				3^rd^ model b			
	B (s.e)	95% CI	t	p-value	B (s.e)	95% CI	t	p-value
**Frailty**								
Frail vs non frail	−2.37 (2.04)	(−6.41, 1.66)	−1.16	0.247	−2.39 (2.05)	(−6.44, 1.66)	−1.17	0.246
Pre-frail vs non-frail	−1.34 (2.00)	(−5.26, 2.65)	−0.65	0.516	−1.57 (2.03)	(−5.59, 2.44)	−0.77	0.440
**Annual personal Income**								
>4500 vs <4500	2.31 (0.80)	(0.72, 3.89)	2.87	0.005	2.30 (0.79)	(0.72, 3.88)	2.88	0.005
**Educational level**								
Highschool vs Uneducated	3.26 (1.12)	(0.83, 5.69)	2.65	0.009	2.94 (1.25)	(0.48, 5.41)	2.36	0.019
Bachelor/MSc/PhD vs Uneducated	4.56 (1.44)	(1.72, 7.39)	3.17	0.002	4.29 (1.45)	(1.43, 7.16)	2.95	0.004
**Age**								
	−0.20 (0.06)	(−0.31, −0.09)	−3.57	<0.001	−0.19 (0.06)	(−0.29, −0.07)	−3.30	0.001
**Gender**								
Men vs women	−0.62 (0.95)	(−2.49, 1.23)	−0.65	0.516	−0.90 (0.96)	(−2.79, 0.99)	−0.94	0.348
**Depression**(GDS)								
severe vs normal	-	-	-	-	−2.61 (1.19)	(−4.97, 0.24)	−2.18	0.031
mild vs normal	-	-	-	-	−1.05 (0.88)	(−2.79, 0.69)	−1.19	0.234
**Comorbitity**								
Severe (CCI≥5) vs mild	-	-	-	-	−0.04 (0.84)	(−1.71, 1.63)	−0.05	0.961

**Example**: In the relation *“Frail vs. non-frail”* it is expected reduction of MoCA score (−2.39 grades), independently of Depression and Comorbidity (adjusted)^b^

**Notes**;

a Linear regression model (2^nd^ model): adjusting for all independent variables;

b (3^rd^ model): adjusting for depression and comorbidity
