# Errata corrige

**Published:** 2019-01-12

The manuscript “Assessing the impact of frailty on cognitive function in older adults receiving home care” published in Transl Med UniSa 2019 Jan 6;19:27–35, reports a mistake in the author list. The correct name for Papathanasiou I is Papathanasiou IV.

