# Supplementary material for: Assessing the Impact of Frailty on Cognitive Function in Older Adults Receiving Home Care
Source: Transl Med UniSa. 2019 Jan 6;19:27–35. (PMC6581500)
Supplement: Supplementary file 1 [file TM-19-027-s001.doc]

Table 1. Demographic data of the participants (n=192)

|  | **Mean ± SD, Median (IQR)** | | |  |
| --- | --- | --- | --- | --- |
| **Age (years)** | 78.04±8.01, 78.00 (12.00) | | |  |
|  | **ν** | **%** | |  |
| **Gender** |  |  | |  |
| Men | 65 | 34.0 | |  |
| Women | 126 | 66.0 | |  |
| **Annual individual Income** |  |  | |  |
| <4500 | 96 | 50.3 | |  |
| >4500 | 95 | 49.7 | |  |
| **Educational level** |  |  | |  |
| Uneducated | 154 | 80.6 | |  |
| Highschool | 21 | 11.0 | |  |
| Bachelor | 15 | | 7.9 | |
| MSc/PhD | 1 | 0.5 | |  |
| **Marital status** |  |  | |  |
| Unmarried | 14 | 7.3 | |  |
| Married | 56 | 29.3 | |  |
| Divorced / Widowed | 121 | 63.4 | |  |
| **Most frequent comorbidities (CCI) ^a^** |  |  | |  |
| Diabetes Mellitus (Type I or II) | 62 | 32.6 | |  |
| Peripheral Vascular Disease | 50 | 26.2 | |  |
| Connective Web Disease | 47 | 24.6 | |  |
| Congestive Heart Failure | 41 | 20.5 | |  |

*Abbreviation:* ***^a^*** *according to* *Charlson Comorbidity Index*
