# Supplementary material for: Assessing the Impact of Frailty on Cognitive Function in Older Adults Receiving Home Care
Source: Transl Med UniSa. 2019 Jan 6;19:27–35. (PMC6581500)
Supplement: Supplementary file 2 [file TM-19-027-s002.doc]

***Table 2. Frequency of the disorders to be investigated (n=192)***

|  | | **ν = 191** | | **%** |  |
| --- | --- | --- | --- | --- | --- |
| **Cognitive Function ^a^** |  | |  | | |
| MoCA <26 | 179 | | 93.7 | | |
| MoCA ≥26 | 12 | | 6.3 | | |
| **Frailty** |  | |  | | |
| Frail | 84 | | 45.9 | | |
| Pre-frail | 91 | | 49.7 | | |
| Non-frail | 8 | | 4.4 | | |
| **Depressiom** |  | |  | | |
| Severe (GDS 11+) | 28 | | 14.7 | | |
| Mild (GDS 6-10) | 82 | | 42.9 | | |
| Normal (GDS 0–5) | 81 | | 42.4 | | |
| **Comorbidity ^b^** |  | |  | | |
| Severe (CCI≥5) | 124 | | 67.8 | | |
| Mild (CCI 2-4) | 59 | | 32.2 | | |
| Normal (CCI 0-1) | 0 | | 0.0 | | |
| **Independence ^c^** |  | |  | | |
| Depentent (Barthel≤10) | 14 | | 7.7 | | |
| Semi-dependent (Barthel11-14) | 22 | | 12.0 | | |
| Independent (Barthel +15) | 147 | | 80.3 | | |
| **Homebound status ^d^** |  | |  | | |
| Homebound | 48 | | 25.1 | | |
| Semi-homebound | 29 | | 15.2 | | |
| Non-homebound | 114 | | 59.7 | | |

***Notes: ^a^MoCA<26*** *indicates cognitive decline;* ***^b^ Comorbidity*** *refers to the mean values of the CCI index and not to the actual number of illnesses;* ***^c^****(****Barthel≤10*** *indicates disability or “disabled” patients);* ***^d^ Homebound status*** *refers to the ability of a person to leave or leaving the home during the last month due to its illnesses.*
