# Supplementary material for: Assessing the Impact of Frailty on Cognitive Function in Older Adults Receiving Home Care
Source: Transl Med UniSa. 2019 Jan 6;19:27–35. (PMC6581500)
Supplement: Supplementary file 3 [file TM-19-027-s003.doc]

***Table 3. Investigation of the impact of frailty and other independent variables on cognitive function (MoCA) [(n = 179]***

| **Independent variables** | **B' (s.e) ^a^** | **95% CI ^b^** | **T** | **p-value** |
| --- | --- | --- | --- | --- |
| **Frailty** |  |  |  |  |
| Frail vs non frail | -5.23 (2.19) | (-9.57, -0.895) | -2.38 | 0.018 |
| Pre- frail vs non- frail | -3.04 (2.19) | (-7.36, 1.29) | -1.38 | 0.168 |
| **Depression** (GDS) |  |  |  |  |
| Severe vs normal | -3.47 (1.23) | (-5.89, -1.04) | -2.81 | 0.005 |
| Mild vs normal | -0.83 (0.89) | (-2.60, 0.95) | -0.92 | 0.359 |
| **Comorbidity** |  |  |  |  |
| Severe (CCI≥5) vs mild | -1.12 (0.94) | (-2.97, 0.73) | -1.19 | 0.233 |
| **Independence** (Barthel) |  |  |  |  |
| Dependent vs independent | -3.37 (1.58) | (-6.50, -0.24) | -2.13 | 0.035 |
| Semi-dependent vs independent | -1.52 (1.35) | (-4.19, 1.15) | -1.12 | 0.262 |
| **Homebound status** |  |  |  |  |
| Homebound vs non- homebound | -3.36 (0.95) | (-5.25, -1.47) | -3.51 | 0.001 |
| Semi- homebound vs non-homebound | -1.41 (1.16) | (-3.69, 0.880) | -1.21 | 0.226 |
| **Cardiovascular diseases (CVDs)** |  |  |  |  |
| Yes vs No | -1.75 (0.84) | (-3.40, -0.10) | -2.09 | 0.038 |
| **Age (years) ^C^** |  |  |  |  |
|  | -0.28 (0.05) | (-0.38, -0.18) | -5.47 | <0.001 |
| **Gender** |  |  |  |  |
| Male vs female | -1.39 (0.92) | (-3.21, 0.42) | -1.51 | 0.132 |
| **Annual individual income** |  |  |  |  |
| >4500 vs <4500 | 2.08 (0.86) | (0.37, 3.78) | 2.41 | 0.017 |
| **Educational level** |  |  |  |  |
| High school vs Uneducated | 3.87 (1.34) | (1.23, 6.52) | 2.89 | 0.004 |
| Bachelor/MSc/PhD vs Uneducated | 5.85 (1.51) | (2.86, 8.84) | 3.86 | <0.001 |

**Abbreviations:** β’ confidence **^a^** (**s.e**): standard error; **^b^ CI**: Confidence Intervals; **c Age**: increase or decrease grades of MoCA per year;

**Note**: MoCA is controlled as deepened variable in this linear model meaning.

**Example**: In the relation *“Frail vs. non-frail”* it is expected reduction of MoCA score (-5.23 grades), this means that as lower scores as greater cognitive function.
