# Supplementary material for: Assessing the Impact of Frailty on Cognitive Function in Older Adults Receiving Home Care
Source: Transl Med UniSa. 2019 Jan 6;19:27–35. (PMC6581500)
Supplement: Supplementary file 4 [file TM-19-027-s004.doc]

Table 4. In-depth analysis for potential confounding effects on association between frailty and cognitive function (n = 179)

| **Independent variables** | **Linear regression model** | | | | **1^st^ model** | | | | |
| --- | --- | --- | --- | --- | --- | --- | --- | --- | --- |
|  | **B (s.e)** | **95% CI** | **t** | **p-value** | **B (s.e)** | **95% CI** | **t** | **p-value** | |
| **Frailty** |  |  |  |  |  |  |  |  | |
| Frail vs non frail | -5.23 (2.19) | (-9.57, -0.895) | -2.38 | 0.018 | -1.89 (2.09) | (-6.03, 2.24) | -0.90 | 0.367 |  |
| Pre frail vs non frail | -3.04 (2.19) | (-7.36, 1.29) | -1.38 | 0.168 | -1.24 (2.03) | (-5.26, 2.77) | -0.61 | 0.542 |  |
| **Depressiom** (GDS) |  |  |  |  |  |  |  |  | |
| Severe vs normal | -3.47 (1.23) | (-5.89, -1.04) | -2.81 | 0.005 | -2.06 (1.27) | (-4.58, 0.45) | -1.62 | 0.107 | |
|
| Mild vs normal | -0.83 (0.89) | (-2.60, 0.95) | -0.92 | 0.359 | -0.94 (0.91) | (-2.74, 0.85) | -1.04 | 0.299 | |
| **Independence** (Barthel) |  |  |  |  |  |  |  |  | |
| Dependent vs independent | -3.37 (1.58) | (-6.50, -0.24) | -2.13 | 0.035 | -1.37 (1.53) | (-4.41, 1.66) | -0.89 | 0.374 | |
|  |
| Semi-dependent vs independent | -1.52 (1.35) | (-4.19, 1.15) | -1.12 | 0.262 | 0.46 (1.29) | (-2.10, 3.03) | 0.35 | 0.721 | |
| **Homebound status** |  |  |  |  |  |  |  |  | |
| Home-bound vs non- homebound | -3.36 (0.95) | (-5.25, -1.47) | -3.51 | 0.001 | -0.49 (1.13) | (-2.73, 1.74) | -0.44 | 0.662 | |
|
| Semi- homebound vs non-homebound | -1.41 (1.16) | (-3.69, 0.880) | -1.21 | 0.226 | 0.21 (1.21) | (-2.18, 2.61) | 0.17 | 0.860 | |
| **Cardiovascular diseases** |  |  |  |  |  |  |  |  | |
| Yes vs No | -1.75 (0.84) | (-3.40, -0.10) | -2.09 | 0.038 | -1.23 (0.81) | (-2.87, 0.32) | -1.58 | 0.115 | |
| **Age (Years)** |  |  |  |  |  |  |  |  | |
|  | -0.28 (0.05) | (-0.38, -0.18) | -5.47 | <0.001 | -0.19 (0.05) | (-0.29, -0.09) | -3.69 | <0.001 | |
| **Income** |  |  |  |  |  |  |  |  | |
| >4500 vs <4500 | 2.08 (0.86) | (0.37, 3.78) | 2.41 | 0.017 | 2.26 (0.78) | (0.71, 3.81) | 2.87 | 0.005 | |
| **Education** |  |  |  |  |  |  |  |  | |
| Highschool vs Uneducated | 3.87 (1.34) | (1.23, 6.52) | 2.89 | 0.004 | 3.19 (1.26) | (0.70, 5.68) | 2.53 | 0.012 | |
| Bachelor /MSc/PhD vs Uneducated | 5.85 (1.51) | (2.86, 8.84) | 3.86 | <0.001 | 4.19 (1.44) | (1.35, 7.03) | 2.91 | 0.004 | |
|

**Example**: In the relation *“Frail vs. non-frail”* it is expected reduction of MoCA score (-1.89 grades), this also means that as lower scores as greater cognitive function
