# Supplementary material for: Assessing the Impact of Frailty on Cognitive Function in Older Adults Receiving Home Care
Source: Transl Med UniSa. 2019 Jan 6;19:27–35. (PMC6581500)
Supplement: Supplementary file 5 [file TM-19-027-s005.doc]

| **Independent variables** | **2^nd^ model ^a^** | | | | | **3^rd^ model ^b^** | | | |
| --- | --- | --- | --- | --- | --- | --- | --- | --- | --- |
|  | | **B (s.e)** | **95% CI** | **t** | **p-value** | **B (s.e)** | **95% CI** | **t** | **p-value** |
| **Frailty** | |  |  |  |  |  |  |  |  |
| Frail vs non frail | | -2.37 (2.04) | (-6.41, 1.66) | -1.16 | 0.247 | -2.39 (2.05) | (-6.44, 1.66) | -1.17 | 0.246 |
| Pre- frail vs non- frail | | -1.34 (2.00) | (-5.26, 2.65) | -0.65 | 0.516 | -1.57 (2.03) | (-5.59, 2.44) | -0.77 | 0.440 |
| **Annual personal Income** | |  |  |  |  |  |  |  |  |
| >4500 vs <4500 | | 2.31 (0.80) | (0.72, 3.89) | 2.87 | 0.005 | 2.30 (0.79) | (0.72, 3.88) | 2.88 | 0.005 |
| **Educational level** | |  |  |  |  |  |  |  |  |
| Highschool vs Uneducated | | 3.26 (1.12) | (0.83, 5.69) | 2.65 | 0.009 | 2.94 (1.25) | (0.48, 5.41) | 2.36 | 0.019 |
|
| Bachelor /MSc/PhD vs Uneducated | | 4.56 (1.44) | (1.72, 7.39) | 3.17 | 0.002 | 4.29 (1.45) | (1.43, 7.16) | 2.95 | 0.004 |
|
| **Age** | |  |  |  |  |  |  |  |  |
|  | | -0.20 (0.06) | (-0.31, -0.09) | -3.57 | <0.001 | -0.19 (0.06) | (-0.29, -0.07) | -3.30 | 0.001 |
| **Gender** | |  |  |  |  |  |  |  |  |
| Men vs women | | -0.62 (0.95) | (-2.49, 1.23) | -0.65 | 0.516 | -0.90 (0.96) | (-2.79, 0.99) | -0.94 | 0.348 |
| **Depression**(GDS) | |  |  |  |  |  |  |  |  |
| severe vs normal | | - | - | - | - | -2.61 (1.19) | (-4.97, 0.24) | -2.18 | 0.031 |
| mild vs normal | | - | - | - | - | -1.05 (0.88) | (-2.79, 0.69) | -1.19 | 0.234 |
| **Comorbitity** | |  |  |  |  |  |  |  |  |
| Severe (CCI≥5) vs mild | | - | - | - | - | -0.04 (0.84) | (-1.71, 1.63) | -0.05 | 0.961 |

**Table 5. Adjusted analysis for factors affecting cognitive function (n = 179).**

**Example**: In the relation *“Frail vs. non-frail”* it is expected reduction of MoCA score (-2.39 grades), independently of Depression and Comorbidity (adjusted)**^b^**

**Notes**; **^a^** Linear regression model (2^nd^ model): adjusting for all independent variables; **^b^**(3^rd^ model): adjusting for depression and comorbidity
